# Clinical efficacy of postauricular injection of Methylprednisolone in the treatment of patients with sudden deafness: A retrospective study

**DOI:** 10.12669/pjms.41.11.13147

**Published:** 2025-11

**Authors:** Xiaoyan Zhu, Jie Li, Min Yang, Tian Zhang, Wandong She

**Affiliations:** 1 Xiaoyan Zhu Department of Otolaryngology, Nanjing Drum Tower Hospital Clinical College of Nanjing University of Chinese Medicine, Nanjing, Jiangsu Province 210008, P.R. China. Affiliated of Nanjing University of Chinese Medicine to Nanjing Integrated Traditional Chinese and Western Medicine Hospital, Nanjing, Jiangsu Province 210008, P.R. China; 2 Jie Li Department of Otolaryngology, Nantong Hospital of Traditional Chinese Medicine, Nantong Hospital Affiliated to Nanjing University of Chinese Medicine, China; 3 Min Yang Department of Otolaryngology, Jiangsu Province Hospital on Integration of Chinese and Western Medicine, Nanjing, Jiangsu Province 210028, P.R. China; 4 Tian Zhang Department of Otolaryngology, Taizhou Hospital of Traditional Chinese Medicine, Taizhou, Jiangsu Province 225300, P.R. China; 5 Wandong She Department of Otolaryngology, Nanjing Drum Tower Hospital Clinical College of Nanjing University of Chinese Medicine, Nanjing 210008, China. Nanjing Drum Tower Hospital, The Affiliated Hospital of Nanjing University Medical School, Nanjing, China, Nanjing, 210008, China

**Keywords:** Sudden deafness, Methylprednisolone, Retroaural injection, Clinical efficacy, Influencing factors

## Abstract

**Objective:**

To assess the clinical effectiveness of postauricular injection of methylprednisolone in the treatment of individuals with sudden deafness (SD) and to identify influencing factors.

**Methodology:**

In this retrospective study, we included clinical data of 106 eligible patients with SD who were hospitalized in the Department of Otorhinolaryngology of Nanjing Integrated Traditional Chinese and Western Medicine Hospital from October 2023 to October 2024. According to the different routes of administration of methylprednisolone, patients were divided into an observation (OBP) (n=54) and a control (CGP) (n=52) group. The CGP group received routine treatment of intravenous methylprednisolone combined with conventional medication and the OBP group received retroauricular injection of methylprednisolone in addition to the routine treatment.

**Results:**

Patients in the OBP group reported a considerably better overall efficacy (87.0% vs. 67.3%) and tinnitus (74.1% vs. 60.7%) than the CGP group. The vertigo disappearance time (5.19±1.29 d vs 5.35±1.06 d) and ear fullness sensation halo disappearance time (5.73±1.21 d vs 6.16±1.27 d) in the OBP group were considerably shorter than in the CGP group (P<0.05). Levels of Homocysteine (HCY), High Shear Blood Viscosity (HBV), Low Shear Blood Viscosity (LBV), Plasma Viscosity (PV) and Hematocrit (HCT) in both groups decreased significantly after therapy and were substantially lower in the OBP group compared to the CGP group (p < 0.05).

**Conclusion:**

Compared to conventional treatment, postauricular injection of methylprednisolone can enhance the treatment efficacy of SD and is associated with improvement in accompanying symptoms. Postauricular injection of methylprednisolone can more effectively reduce HCY, HBV, LBV, PV and HCT levels in SD patients.

## INTRODUCTION

Sudden deafness (SD) typically refers to the sudden and inexplicable onset of sensorineural hearing loss affecting at least two consecutive frequencies and greater than 20 dB, which occurs within 72 hours, most commonly in unilateral ears and rarely in both ears simultaneously.^1^,^2^ In addition to hearing loss symptoms, SD patients may report a series of clinical symptoms such as tinnitus, ear tightness, dizziness, and even vomiting.^3^ In some cases, SD is accompanied by anxiety, depression and other manifestations.^4^ At present, the etiology and pathogenesis of SD are not completely clear. The more recognized factors include viral infection, inner ear microcirculation disorder, immune factors and metabolic disorders.^5^-^8^ The treatment methods of SD mainly focus on the application of glucocorticoids (GC), such as methylprednisolone, as well as vasodilators, neurotrophins, thrombolytics. However, while timely treatment relieves some of the symptoms, the effect is not ideal.^9^,^10^

For SD patients, the GC treatment route generally includes systemic, tympanic and postauricular administration.^11^ Systemic use of GC is usually seen as a routine treatment. However, due to the blood-labyrinth barrier, achieving an effective therapeutic concentration of systemically administered GC in the inner ear often requires a large dose, which is associated with numerous adverse reactions and limits its use in patients with GC contraindications.^12^,^13^

Postauricular GC administration has gradually attracted clinical attention. This method can be used both for the first onset of SD and as a remedial treatment after the failure of conventional therapy or an alternative treatment for SD patients with hormone contraindications such as hypertension, diabetes and peptic ulcer.^14^,^15^ Additionally, regional studies have provided valuable insights into the clinical patterns and treatment strategies of SSNHL in South Asian populations. Recent data from Pakistan suggest similar demographic distributions and highlight the role of early steroid intervention in improving auditory outcomes.^16^,^17^

Multiple studies have reported the clinical efficacy of postauricular steroid injection for sudden sensorineural hearing loss (SSNHL). For instance, Gao et al.^18^ and Chen et al.^19^ observed improved auditory outcomes and faster symptom resolution using postauricular methylprednisolone compared to systemic therapy. These findings are in line with our results, which demonstrated significantly better hearing recovery and symptom improvement in the observation group. Moreover, the postauricular route has been recognized as a minimally invasive and well-tolerated approach, potentially delivering higher local steroid concentrations in the cochlear fluids via vascular or lymphatic diffusion. Compared to the intratympanic route, it avoids complications such as tympanic membrane perforation, and compared to intravenous therapy, it results in fewer systemic effects - a benefit especially relevant for elderly patients or those with comorbidities.

Nonetheless, prior studies also highlight several limitations, including heterogeneity in dosing regimens, variable definitions of treatment response, and the lack of long-term follow-up data. These issues also apply to our study, and we emphasize the need for future multicenter trials with standardized protocols and pharmacokinetic analysis to clarify the optimal use of this method.^18^,^19^ This study aimed to examine the clinical efficacy of postauricular injection of methylprednisolone combined with systemic administration in managing SD patients and to identify potential factors influencing treatment effectiveness.

## METHODOLOGY

This retrospective study included 106 patients with sudden deafness who were hospitalized in the Department of Otorhinolaryngology of Nanjing Integrated Traditional Chinese and Western Medicine Hospital from October 2023 to October 2024. As this was a retrospective clinical study, the division of patients into the observation group (OBP) and the control group (CGP) was based on their received treatment regimen documented in the hospital records between October 2023 to October 2024. The treatment allocation was not randomized, but instead reflected clinical decisions made by the attending physicians at the time. Moreover, no blinding was applied, as the data were collected retrospectively from existing electronic medical records after treatments had already been administered. The CGP group (n=52) received routine treatment that included intravenous methylprednisolone combined with conventional medication and the OBP group received retroauricular injection of methylprednisolone in addition to the routine treatment. All patients completed the otolaryngology specialist examination, which included pure tone audiometry, acoustic impedance examination, otoendoscopy and a magnetic resonance imaging scan of the internal auditory canal. Patients had also undergone routine examinations, including hematuria and fecal tests, biochemistry, a chest X-ray and an electrocardiogram.

### Ethical Approval:

The Ethics Committee of Nanjing Integrated Traditional Chinese and Western Medicine Hospital approved the study (Approval number 2022024); Date: February 22, 2022. Due to the retrospective nature of the study, informed consent was waived.

### Inclusion criteria:

- Diagnosis of sudden sensorineural hearing loss in accordance with the “Guidelines for the Diagnosis and Treatment of Sudden Deafness” by the Chinese Medical Association.^1^
- Admission to hospital within seven days of symptom onset.
- Unilateral involvement.
- No prior use of glucocorticoids, vasodilators, or any treatments for hearing loss prior to admission.
- No history of Meniere’s disease, middle or inner ear inflammation, or other known etiologies of hearing loss.
- No family history of deafness.

### Exclusion criteria:

- Pregnancy or lactation.
- Hearing loss due to known causes such as otitis externa, otitis media, central nervous system diseases, trauma, ototoxic drugs, or noise exposure.
- Presence of systemic diseases such as hypertension, diabetes, or chronic cardiovascular conditions, which may confound the treatment response.
- Patients with incomplete clinical data or inability to evaluate treatment efficacy.

### Treatment plans:

### Routine treatment:

Methylprednisolone Sodium Succinate for Injection (Guoyao Group Rongsheng Pharmaceutical Co., Ltd., H20040844) was administered as follows: 80mg for four days 40mg for four days 20mg for two days. Pentoxifylline 100mg (Jiuquan Dadeli Pharmaceutical Co., Ltd., H20031148) was intravenously injected for 10 days and mecobalamin 0.5mg (Chenxin Pharmaceutical Co., Ltd., H20055734) was intravenously injected for 10 days.

### Retroauricular methylprednisolone:

In addition to the routine regimen, Methylprednisolone Sodium Succinate (20 mg) + 1 ml of lidocaine hydrochloride (Hubei Tiansheng Pharmaceutical Co., Ltd., H42021839) was injected under the periosteum of the ear, once a day for a total of 10 times. Both groups were treated continuously for 10 days and then continued to take mecobalamin (North China Pharmaceutical Co., Ltd., H20031126) 0.5 mg three times a day and ginkgo biloba leaves (Yangtze River Pharmaceutical Group Co., Ltd., Z20027949) 19.2 mg three times a day for one month.

### Observation indicators:

The following data were collected from all patients:

- General characteristics, such as name, gender, age of onset, side of the affected ear.
- Accompanying symptoms, including tinnitus, ear fullness and dizziness.
- Clinical data. The severity of hearing loss was detected using the pure tone audiometer (Otometrics, Aster1066) and the type of hearing loss curve was detected by acoustic immittance meter (Otometrics, 1096SA).
- Clinical efficacy, including disappearance time of vertigo symptoms and ear fullness, improvement of accompanying symptoms such as tinnitus, ear fullness and vertigo. Clinical hearing efficacy and tinnitus efficacy were evaluated at the end of treatment.
- Laboratory indicators were measured in 5-mL of fasting venous whole blood before and after treatment. High shear blood viscosity (HBV), low shear blood viscosity (LBV), plasma viscosity (PV) and hematocrit (HCT) were measured by an automatic blood rheology tester (Seckheed, SUCCEEDER SA-7000). LABOSPECT 008 AS automatic biochemical analyzer (Hitachi, Inc) was used to detect serum Homocysteine (HCY) level by cyclic enzymatic method.

### Clinical efficiency evaluation:

### Hearing loss severity grading standard:

According to the grading standard of pure tone hearing threshold of average language frequency, 250Hz, 500Hz, 1000Hz, 2000Hz, 3000Hz, 4000Hz, 8000Hz were included in the calculation range. Taking the hearing loss of single ear as the standard, the individuals’ pure tone hearing threshold test results were sorted out and then the degree of hearing loss was graded according to the following standards:

- Normal: average hearing threshold ≤ 25dBHL.
- Mild: average hearing threshold 26-40 dBHL.
- Moderate: the average hearing threshold 41-55 dBHL.
- Moderate to severe: the average hearing threshold 56-70 dBHL.
- Severe: the average hearing threshold 71-90 dBHL.
- Extremely severe: average hearing threshold > 90dBHL.

### Hearing loss curve type classification standard:

By estimating the pure tone hearing threshold test results of the subjects and according to the frequency of hearing loss, the hearing loss curve was classified. The types of hearing loss included:

- Low-frequency hearing loss: hearing loss at frequencies below 1000 Hz (inclusive) and at least 250 and 500 Hz > 20 dBHL are two examples.
- High frequency type: hearing loss < 20dBHL at least 4000 and 8000 Hz and hearing loss below 2000 Hz (included).
- Flat decline type: all frequencies of hearing loss, 250~8000Hz average hearing threshold ≤ 80dBHL.
- Total deafness: hearing loss across all frequency ranges, with an average hearing threshold of > 80 dBHL at 250-8000 Hz.

### Hearing efficacy evaluation criteria:

According to the grading criteria for the efficacy of sudden deafness in the guidelines, the pure tone audiometry values reviewed by the patients were divided as follows:

- Cured: the hearing of the damaged frequency returned to normal, or reached the level of the healthy ear, or reached the level before the disease.
- Markedly effective: the average hearing loss frequency increased by more than 30 dBHL.
- Effective: the average hearing loss frequency increased by 15 ~ 30 dBHL.
- Ineffective: the average hearing improvement of the damaged frequency was less than 15 dBHL.
- ***Tinnitus curative effect judgment standard:*** Based on the ‘ Eye, Otorhinolaryngology Disease Diagnosis and Treatment Guidelines and Nursing ‘, combined with the severity of tinnitus before and after medication, the efficacy of tinnitus was divided into the following:
- Cured: tinnitus is completely eliminated.
- Markedly effective: the grade of tinnitus was reduced by at least two grades.
- Effective: tinnitus grade decreased by one grade.
- Ineffective: the degree of tinnitus did not change or even increased.

This grading method is widely adopted in Chinese otolaryngology clinical practice; however, it is not a globally standardized assessment tool and is based on subjective symptom reporting rather than a validated tinnitus scale. The total effective rate (clinical efficiency evaluation index) was calculated as the sum of the cured rate, the markedly effective rate and the effective rate.

### Statistical analysis:

The data was analyzed using the statistical program SPSS26.0. The measurement data was presented as mean ± standard deviation. The difference between the two groups was assessed using the t-test, while the differences between multiple groups were examined using analysis of variance. The count data were reported as frequencies and rates, with the chi-square test employed to compare the groups. Logistic regression analysis was utilized to identify the characteristics that influence effectiveness. Results were presented as odds ratios (OR) with 95% confidence intervals (CI). Statistically significant differences were defined as P < 0.05.

## RESULTS

As shown in Table-I, there was also no discernible difference between the two groups in terms of baseline information and clinical data (P>0.05). Among the 54 patients in the OBP group, 27 reported tinnitus, with a total efficacy of 74.1 %, considerably higher than in the CGP group (60.7%; P<0.05). The OBP group reported a superior overall hearing efficacy rate, 87.0%, compared to the 67.3% of the CGP group (P<0.05). Table-II. The average time to disappearance of vertigo and ear fullness in the OBP group was significantly shorter than in the CGP group (P<0.05).

**Table-I:** General data and clinical features of patients in the two groups.

Parameter	CGP (n=52)	OBP (n=54)	t/x^2^	P
***Gender***			0.053	0.818
Male	21	23		
Female	31	31		
Age	44.6±10.91	43.72±10.64	0.146	0.677
***Ear side***			0.344	0.557
Left	24	28		
Right	28	26		
***Concomitant symptom***				
No	10	12	0.144	0.704
***Tinnitus***			0.354	0.552
Yes	29	27		
No	23	27		
***Dizziness***			0.036	0.894
Yes	26	26		
No	26	28		
***Ear fullness***			1.401	0.236
Yes	31	26		
No	21	28		
***Hearing loss severity***			6.912	0.141
Mild	3	3		
Moderate	19	15		
Moderate to severe	5	9		
Severe	12	16		
Extremely severe	13	11		
***Type of hearing loss curve***			0.922	0.82
Low-frequency descending type	4	5		
High frequency descending type	12	15		
Flat descending type	11	13		
All deaf type	25	21		

**Table-II:** Comparison of clinical efficacy between the two groups.

Parameter	CGP (n=52)	OBP (n=54)	t/x^2^	P
***Tinnitus effect:***			7.868	0.049
Healed	1	7		
Significantly effective	8	3		
Effective	8	10		
Invalid	11	7		
Total effective rate	60.7%	74.1%	6.734	0.009
***Hearing effect:***				
Healed	8	11		
Significantly effective	12	21		
Effective	15	15		
Invalid	17	7		
Total effective rate	67.3%	87.0%	5.887	0.015
The disappearance time of vertigo symptoms	5.73±1.21	5.19±1.29	2.221	0.029
Disappearance time of ear fullness sensation	6.16±1.27	5.35±1.06	0.673	0.021

**Fig.1:**
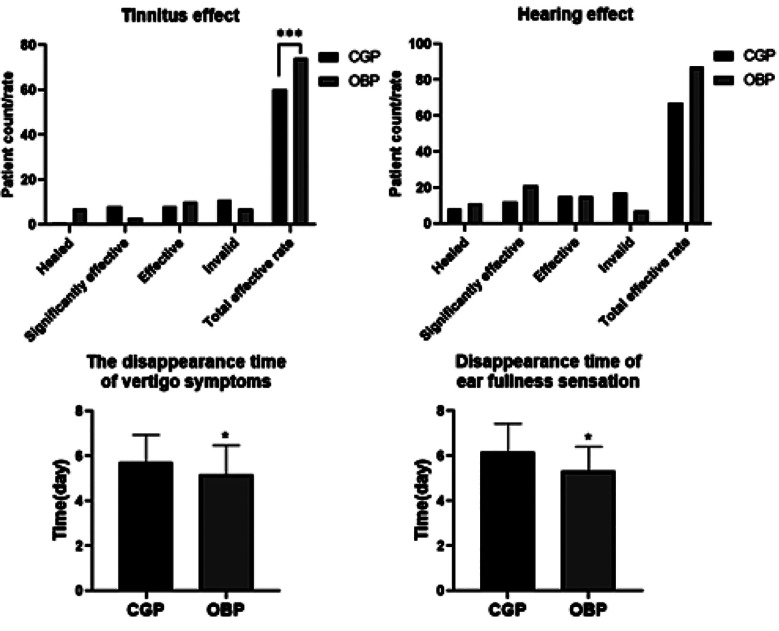
Comparison of clinical efficacy between the two groups.

**Fig.2:**
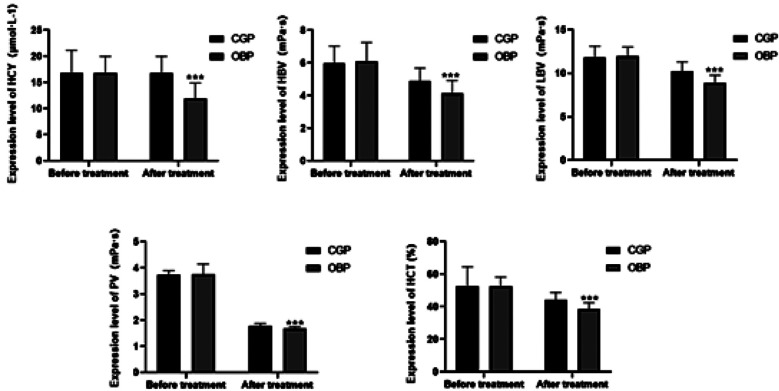
Comparison of laboratory indicators before and after treatment between the two groups.

Prior to treatment, HCY, HBV, LBV, PV and HCT levels of both groups were comparable (Table-III). Following therapy, there was a substantial reduction in the levels of HCY, HBV, LBV, PV and HCT in both groups (P<0.05) and the OBP group reported markedly lower post-treatment levels of these indices compared to the CGP group. (Fig.1 and Table-III), P<0.05. The independent variable and the patients’ general data as the dependent variable, gender was identified as a factor influencing effectiveness (Fig.2).

**Table-III:** Comparison of laboratory indicators before and after treatment between the two groups.

Parameter	CGP (n=53)	OBP (n=54)	t	P
HCY before treatment (μmol·L^-1^)	16.87±4.31	16.81±3.21	10.867	0.937
HCY after treatment (μmol·L^-1^)	14.92±4.36	11.88±3.02	2.322	<0.001
HBV before treatment (mPa·s)	5.99±1.04	6.1±1.16	1.656	0.628
HBV after treatment (mPa·s)	4.92±0.78	4.15±0.79	0.426	<0.001
LBV before treatment (mPa·s)	11.84±1.28	11.97±1.05	0.295	0.565
LBV after treatment (mPa·s)	10.23±1.08	8.91±0.87	1.082	<0.001
PV before treatment (mPa·s)	3.74±0.16	3.76±0.39	32.016	0.776
PV after treatment (mPa·s)	1.78±0.1	1.7±0.06	4.36	<0.001
HCT before treatment (%)	52.66±11.83	52.47±5.79	34.723	0.916
HCT after treatment (%)	44.06±4.77	38.7±3.83	5.962	<0.001

When clinical features were used as the dependent variable and effectiveness as the independent variable, vertigo, ear fullness and the kind of hearing loss curve were identified as influencing factors. In contrast, the effectiveness was unaffected by the degree of hearing loss or tinnitus.

## DISCUSSION

This study demonstrated that in SD patients, postauricular injection of GS, combined with the routine treatment regimen (intravenous steroids combined with conventional treatment), is associated with a greater overall hearing and tinnitus efficacy compared to the routine treatment alone. Furthermore, the combined regimen was linked to a shorter time to disappearance of vertigo and ear fullness. As an otology emergency, SD may lead to hearing loss and, in severe cases, full deafness, which significantly impacts quality of life and is associated with serious psychological burden.^20^ At present, GC treatment is considered standard care for the treatment of SD.

Our findings are consistent with studies conducted in South Asian settings. For instance, Sajid et al. and Naz et al. reported favorable outcomes with corticosteroid-based therapy in SD patients, particularly when initiated within the acute window.^16^,^17^ These results further support the applicability of postauricular steroid administration as a viable alternative in resource-constrained environments.

Studies show that GCs, such as methylprednisolone, act by binding to specific receptors widely distributed in the inner ear, inhibiting the body’s immune response, improving the microcirculation of the inner ear and restoring and maintaining the balance and stability of potassium and sodium ions in the endolymph, ultimately reducing the hydrops of the membrane.^21^,^22^ Among GCs used in clinical practice, methylprednisolone has minimal effect on glucose metabolism and easily crosses the blood-labyrinth barrier, making it a GC of choice for treating SD.^23^-^25^ In the past, systemic administration was often used in the treatment of SD with GC; however, achieving an effective drug concentration often necessitates increasing the dosage and prolonging the medication time, which would increase the related adverse reactions.^14^,^26^ With the recent research on the microcirculation of the inner ear, the local administration of GC by postauricular injection has become more popular.^27^

Recent studies suggest that postauricular injection of methylprednisolone may facilitate targeted drug delivery to the inner ear by partially bypassing the blood–labyrinth barrier (BLB). Following subperiosteal administration behind the ear, the corticosteroid can be absorbed via the retroauricular venous plexus and the stylomastoid artery, potentially reaching the endolymphatic sac and perilymph through vascular and lymphatic pathways, such as the cochlear aqueduct and periauricular lymphatics.^28^ Compared with conventional intravenous administration, which requires higher systemic doses to overcome the BLB, the postauricular route enables higher local drug concentrations in the cochlea and vestibular system with significantly reduced systemic exposure. This pharmacokinetic advantage translates into a faster onset of action and a lower incidence of systemic adverse effects, making it a safer and more targeted alternative-particularly for patients with comorbidities like hypertension or diabetes.^29^ Moreover, unlike intratympanic injections, the postauricular method avoids direct middle ear manipulation and related complications such as tympanic membrane perforation, while still achieving favorable therapeutic outcomes. Based on emerging pharmacological and anatomical evidence, postauricular injection offers a promising drug delivery strategy for the treatment of sudden sensorineural hearing loss SD.

As the blood supply of the inner ear of SD patients is insufficient, there is a phenomenon of slow, stagnant and blocked blood flow, which leads to the abnormality of hemorheology.^30^ This study found high pre-treatment levels of HCY, HBV, LBV, PV and HCT in all patients, consistent with the previous results.^3^,^31^ The clinical significance of HCY, HBV, and LBV in SSNHL lies in their potential contribution to inner ear microcirculatory disturbances.^32^ Elevated homocysteine (HCY) is known to promote oxidative stress, impair endothelial function, and increase blood coagulability, all of which may compromise cochlear perfusion.^33^ Likewise, increased blood viscosity, reflected by higher HBV and LBV values, can lead to impaired capillary flow, elevated vascular resistance, and local ischemia in the cochlear microvasculature.^34^ These hemodynamic abnormalities are consistent with one of the leading hypotheses in SD pathogenesis-that sudden hearing loss is caused by transient or sustained ischemia of the inner ear. Therefore, these laboratory indices not only serve as markers of systemic vascular status but may also provide indirect evidence of cochlear hypoperfusion, allowing clinicians to evaluate both disease severity and therapeutic response more effectively.

The defect also increases the likelihood of thrombus formation in the common cochlear artery of patients with high homocystein, which can cause severe damage to the hair cells and microcirculation ischemia, further aggravating the symptoms of hearing loss.^35^ In addition, the increased expression of HCY has been linked to endothelial cells damage, increased platelet aggregation and adhesion rate and changes in the coagulation-fibrinolysis system, leading to blood hypercoagulability and microcirculation disorders in the inner ear.^36^ Abnormal HCY metabolism is indeed considered to be closely related to sudden deafness.^34^

Methylprednisolone acts by dilating spasmodic blood vessels, inhibiting platelet activation, increasing myocardial contraction and preventing microthrombosis, significantly improving the blood oxygen supply of the inner ears.^35^,^37^ Thus, hemorheology and homocysteine levels may have a prognostic value in patients with idiopathic deafness. In this study, the levels of HCY, HBV, LBV, PV and HCT in the two groups were significantly decreased after treatment and the decrease was markedly greater in the OBP group, confirming that retroauricular injection of methylprednisolone is more effective in improving patients’ blood rheology status, inner ear ischemia and hypoxia.^38^,^39^ In addition, multivariate analyses revealed that HCY, HBV and HCT levels were independent risk factors affecting the efficacy of the combined treatment regimen, further confirming the importance of optimizing HCY, HBV and HCT levels in the treatment of SD. These results suggest that SD patients would benefit from monitoring the coagulation function, HCY level and hematocrit as reference indicators for therapeutic effect.

This study presents several notable strengths. First, it is one of the few retrospective analyses that systematically evaluates the combination therapy of postauricular methylprednisolone injection and conventional intravenous administration in patients with sudden sensorineural hearing loss SD. Second, the study comprehensively incorporates both audiological and hemorheological parameters as outcome measures, providing a multidimensional assessment of treatment efficacy. Third, the application of multivariate logistic regression analysis enhances the robustness of the conclusions by identifying independent predictors of clinical response.

Lastly, the study offers a detailed stratified analysis of tinnitus-related outcomes, which adds to the clinical relevance. However, certain limitations remain and should be addressed in future research. These include the retrospective design, limited sample size, and lack of long-term follow-up. Future directions include conducting multicenter, prospective randomized controlled trials (RCTs) with standardized postauricular injection protocols, exploring the pharmacokinetics and molecular mechanisms of steroid delivery via the postauricular route, incorporating validated tinnitus scales, and assessing the durability of hearing improvement over extended periods. These steps will help to substantiate the clinical utility and mechanistic understanding of postauricular steroid therapy in the management of SD.

### Limitations:

First, due to its retrospective, single-center design and relatively small sample size, the generalizability of our findings is limited. Treatment allocation was based on clinical judgment rather than randomization, and neither blinding nor control conditions were applied, which may introduce selection and information biases. Second, although we applied strict inclusion and exclusion criteria to ensure population homogeneity, residual confounding cannot be completely ruled out. Factors such as patient lifestyle, medication history, psychosocial status, and treatment adherence were not consistently recorded in medical files and thus not included in the multivariate regression analysis. These uncontrolled variables may influence outcomes and reduce the internal validity of our findings. Third, the absence of long-term follow-up is a notable limitation.

SD can exhibit delayed or fluctuating therapeutic responses, and the potential for symptom recurrence or delayed adverse reactions-particularly with novel delivery methods like postauricular injection-remains a clinical concern. Our analysis was confined to short-term outcomes due to incomplete follow-up records. Future longitudinal cohort studies are warranted to assess long-term efficacy, relapse rates, and delayed complications. Lastly, the assessment of tinnitus was based on a semi-quantitative grading system, which, while commonly used in our clinical setting, lacks standardization. Future research should adopt internationally validated tools such as the Tinnitus Handicap Inventory (THI) or the Tinnitus Severity Scale (TSS) to improve assessment consistency and comparability. To address these limitations, future studies should adopt multicenter, prospective, randomized controlled designs with comprehensive data collection and standardized outcome measures. This will improve both the internal and external validity of the findings and provide more robust evidence for the clinical utility of postauricular methylprednisolone therapy in SD.

## CONCLUSION

This study demonstrates that postauricular injection of methylprednisolone, when combined with systemic glucocorticoid therapy, provides superior treatment efficacy for sudden deafness (SD) compared to systemic administration alone. The combined approach also significantly reduces hemorheological parameters such as HCY, HBV, LBV, PV, and HCT. Furthermore, HCY, HBV, and HCT were identified as independent risk factors influencing treatment outcomes, highlighting their potential prognostic value.

However, these findings should be interpreted with caution due to the retrospective, single-center design and the limited sample size, which may restrict the generalizability of the results to broader populations. Additionally, our analysis was confined to short-term outcomes, and long-term efficacy, recurrence rates, and delayed adverse effects of the combined regimen remain unclear. Future research should focus on prospective, multicenter randomized controlled trials with extended follow-up durations to validate our findings, reduce potential biases, and better assess the long-term safety and effectiveness of postauricular methylprednisolone therapy in SD.

### Funding:

Nanjing Municipal Health Science and Technology Development Program (No. YKK22185); Jiangsu Province Traditional Chinese Medicine Classical Prescriptions Evaluation and Transformation Engineering Research Center Open Topics (No. GCZX-20240111); Jiangsu Provincial Project for the Development of Traditional Chinese Medicine Science and Technology (No.MS2024049). Natural Science Foundation of Nanjing University of Traditional Chinese Medicine (XZR2024122).

### Authors’ contributions:

**XZ:** Study design, literature search and manuscript writing.

**JL, MY, TZ and WS:** Data collection, data analysis and interpretation. Critical review.

**XZ:** Manuscript revision and validation and is responsible for the integrity of the study.

All authors have read and approved the final manuscript.
